# CD137 ligand feedback upregulates PD‐L1 expression on lung cancer via T cell production of IFN‐γ

**DOI:** 10.1111/1759-7714.13207

**Published:** 2019-10-17

**Authors:** Helin Wang, Zhuohong Yan, Jianqing Hao, Bin Yang, Jinghui Wang, Ling Yi, Xiaojue Wang, Shuping Li, Hongtao Zhang, Shucai Zhang

**Affiliations:** ^1^ Department of Oncology, Beijing Chest Hospital Capital Medical University, Beijing Tuberculosis and Thoracic Tumor Research Institute Beijing China; ^2^ Central Laboratory, Beijing Chest Hospital Capital Medical University, Beijing Tuberculosis and Thoracic Tumor Research Institute Beijing China

**Keywords:** CD137 ligand, IFN‐γ, immune evasion, lung cancer, PD‐L1

## Abstract

**Background:**

The expression of PD‐L1 and its regulation in tumors remains unclear. The importance of IFN‐γ in upregulating the PD‐L1 expression in various tumors, and the effects of other essential cytokines in the tumor microenvironment (TME), need to be further elucidated.

**Methods:**

Constitutive expression of PD‐L1 and CD137L in all 13 lung cancer cell lines were tested by flow cytometry. CD137L mRNA of lung cancer cell lines was detected by RT‐PCR. PD‐L1 expression rates following stimulation with these cytokines (IFN‐γ, TNFα and IL2) were measured. After coculture of cells expressing CD137L (lung cancer cells or 293FT cells transfected with CD137L plasmid) with T cells, the PDL1 expression of lung cancer cells and IFN‐γ in supernatant was detected.

**Results:**

Our data revealed that adenocarcinoma and squamous cell carcinoma cells had the highest positive expression rate. IFN‐γ was the core‐inducing factor for enhancing the PD‐L1 expression. CD137L was also widely expressed in the lung cancer cell lines at the mRNA level, whereas its expression was generally low at the protein level. However, the low expression of CD137L protein was still enough to induce T cells to produce IFN‐γ, which subsequently increased the PD‐L1 expression by lung cancer cells. The CD137 signal induces IFN‐γ secretion by T cells, which stimulates high‐level of PD‐L1 expression in cancer cells; this negative immune regulation may represent a mechanism of immune escape regulation.

**Conclusions:**

CD137L mRNA was widely expressed in lung cancer cell lines whereas levels of protein expression were generally low. The low level of CD137L protein was still enough to induce T cells to produce IFN‐γ that subsequently increased PD‐L1 expression. The CD137L‐induced negative immune regulation may represent a mechanism of immune escape.

## Introduction

The PD‐1/PD‐L1 pathway plays a leading role in tumor escape from immune responses.^1^ Immune checkpoint inhibitors abolish the inhibitory signal of T cell activation, allowing tumor‐reactive T cells to overcome regulatory mechanisms and produce potent antitumor responses.^2^, ^3^, ^4^ Pembrolizumab, an inhibitor of PD‐1/PD‐L1, was first approved for therapeutic use in 2014 and is currently available for treating various tumor types.^5^ Among them, non‐small cell lung cancer is the strongest indication for this therapy. However, only a few of patients benefit from this treatment. Therefore, identifying biomarkers that can predict patient response has become a top priority.^6^

It has been proposed that tumor cell PD‐L1 plays a significant role in impeding T lymphocyte‐mediated killing. PD‐L1 is an important factor for poor prognosis in patients with lung adenocarcinoma.^7^ Furthermore, PD‐L1 higher expression of carcinoma cells has been shown to be connected with a better immunological response after checkpoint blockade. Thus, the PD‐L1 expression on cancer cells plays a large role in predicting the therapeutic effect and patient prognosis of immune checkpoint block therapy.^8^, ^9^, ^10^, ^11^

PD‐L1 expression can be constitutive or inducible. In carcinogenesis, PD‐L1 can be overexpressed as a result of driver oncogenic events.^12^ Of note, it has previously been reported that a variety of cytokines could induce PD‐L1 expression, such as interferon gamma (IFN‐γ),^13^ tumor necrosis factor alpha (TNF‐α),^14^ interleukin 17 (IL‐17),^15^ IL‐10,^16^ IL‐1β,^17^ IL‐6,^18^ and IL‐27.^19^ In particular, further work on the potential role of IFN‐γ is needed because this cytokine is significant for blocking PD‐1/PD‐L1 clinically and could lead to the development of related combination therapy.

CD137, a representative costimulatory molecule belong to TNF‐receptor superfamily, is an important target for tumor immunotherapy.^20^ Its gene expression is inducible in activated T cells.^21^ In early reports, monoclonal antibodies (mAbs) against CD137 have been shown to preferentially induce CD8 T cell proliferation compared to CD4 T cells and to eradicate established tumors in a mouse model.^21^, ^22^ Stimulating CD137 in vivo was found to alter the trafficking of CD8^+^ T lymphocytes and elevate the production of IFN‐γ and TNF‐α. These events were dependent on increasing CD8 T lymphocytes.^22^, ^23^ Expression of CD137 ligand (CD137L) has been found on activated T cells, B cells, dendritic cells, and macrophages.^24^, ^25^ Furthermore, CD137L is constitutively expressed on cancer cell lines, and, when T cells were cocultured with carcinoma cells, the expression of CD137L resulted in higher levels of IFN‐γ production by the activated T cells.^26^

Although the antitumor effect of CD137 mAb has been widely recognized, there are still cases of treatment failure. Treatment with an anti‐CD137 antibody that was associated with inducing PD‐L1 expression in tumors was found to be ineffective for curing tumors in a mouse model.^9^ Additionally, multiple studies have shown a powerful synergy between anti‐CD137 mAb and anti‐PD‐1/PD‐L1 mAb when they are applied in combination.^27^, ^28^, ^29^ Here, we focused on lung cancer cell lines and investigated whether CD137L could upregulate PD‐L1 expression by lung cancer cells via feedback involving IFN‐γ production by activated T lymphocytes.

## Methods

### Cell preparation

Human T lymphocytes were derived from EDTA anticoagulated peripheral blood from healthy donors. Peripheral blood mononuclear cells were isolated by Ficoll‐Paque PLUS (GE Healthcare Life Sciences, Uppsala, Sweden). T lymphocytes labeled with antihuman CD3 microbeads (Miltenyi Biotec Inc., Bergisch Gladbach, Germany) were sorted using a Miltenyi mini kit (Miltenyi Biotec Inc.). The human lung cancer cell lines and 293FT cells were purchased from the Cell Resource Center of the Institute of Basic Medical Sciences of the Chinese Academy of Medical Sciences. Adenocarcinoma cell lines(A2, A549, NCI‐H2009, HCC‐827, CALU‐1), squamous cell carcinoma cell lines (NCI‐H2170, NCI‐H1703), large cell carcinoma cell lines (PLA‐801D, NCI‐H460, NCI‐H661) and small cell carcinoma cell lines (NCI‐H446, NCI‐H69) were all cultured in 10% FBS/RPMI‐1640 medium. NCI‐H209 (small cell carcinoma) was cultured in 20% FBS/RPMI‐1640 medium. 293FT cells were cultured in 10% FBS/DMEM medium. They were all cultured at 37°C in 5% CO_2_. The study was approved in advance by healthy donors consent and the Ethics Committee of Beijing Chest Hospital affiliated to Capital Medical University.

### 293FT cell transfection

To express CD137L on the surface of 293FT cell membranes, the cDNA encoding the extracellular domain of CD137L was transferred into the 293FT cytoplasm via the vector pCMV3‐C‐GFPSpark (Sino Biological Inc., Beijing, China) and the lipofectamine 2000 reagent (Invitrogen, Carlsbad, CA, USA) according to the manufacturer's instructions. After transfection, the cells were cultured in 10% FBS/DMEM medium for 48 hours. The positive rate of transfection was obtained by flow cytometry.

### Antibody and proteins

Antihuman PD‐L1 mAb (clone MIH1), mouse IgG1, κ isotype control (clone X40), antihuman CD137L (clone C65‐485) and antihuman CD137 mAb (clone h41BB‐M127) were purchased from BD Biosciences (San Jose, CA, USA). Goat antimouse IgG polyclonal Ab was purchased from Abcam (Cambridge, MA, USA). Human TNF‐α protein, TNF‐β protein, IL2 protein, IFN‐γ protein and human CD137 protein (with the Fc region of human IgG1) were purchased from Sino Biological Inc. Rabbit Antihuman IgG polyclonal Ab was purchased from Bioss (Tongzhou, Beijing, China). Antihuman CD3 mAb (clone OKT3) was purchased from Miltenyi Biotec Inc.

### Flow cytometry

The membrane PD‐L1 or CD137L expression of lung cancer cell lines was detected via flow cytometry. The cells were stained with PE‐CF594‐labeled antihuman PD‐L1 mAb, PE‐labeled antihuman CD137L mAb, or PE‐CF594‐labeled mouse IgG1, κ isotype control. To obtain enhanced signals of PD‐L1, the cells were stained with mouse antihuman PD‐L1 mAb or mouse IgG1, κ isotype control, followed by staining with a secondary DyLight 488‐conjugated goat antimouse polyclonal Ab. To obtain intensive signals of CD137L, the cells were stained with human CD137 protein (with the Fc region of human IgG) or human B7‐2Ig, followed by staining with a secondary APC‐conjugated rabbit antihuman IgG polyclonal Ab. Analytical gates were established using isotype, fluorescent matching and nonspecific monoclonal antibodies as controls. Lung cancer cell lines were treated with IFN‐γ (100 ng/mL), TNF‐α (50 ng/mL), TNF‐β (10 ng/mL), IL‐2 (100 U/mL) for 72 hours, respectively. PD‐L1 or CD137L expression was then detected. FlowJo software (Tree Star V10, Ashland, OR, USA) was utilized for flow cytometric data analysis. The mean fluorescence intensity (MFI) of the same pathological type before or after induction of IFN‐γ was averaged.

### RNA extraction

The total RNA of the lung cancer cell lines was extracted with RNAprep Pure Cell Kit (Tiangen Biotech, Beijing, China) according to the manufacturer's instructions. cDNA was then synthesized at 42°C for 15 minutes followed by heating to 95°C for three minutes to establish a library according to the instructions provided in the FastQuant cDNA First Chain Synthesis Kit (Tiangen Biotech).

### RT‐PCR

The expression of CD137L mRNA in lung cancer cell lines was detected using RT‐PCR. To amplify human CD137L cDNA, the following primers were used: sense primer, 5′ ATT ACC GGT GCA GCC TGC CCC TGG GCC GTG‐3′; and antisense primer, 5′‐ATT GGA TCC TTC CGA CCT CGG TGA AGG GAG‐3′. The housekeeping gene β‐actin served as a control: sense primer, 5′‐CCC AGA TCA TGT TTG AGA CCTT‐3′; antisense primer, 5′‐GTG GTG GTG AAG CTG TAG CC‐3′. After denaturation at 95°C for five minutes, 35 cycles of PCR were performed, each consisting of a denaturation step (94°C for 30 seconds), an annealing step (62°C for 30 seconds), and an elongation step (72°C for 30 seconds), followed by a final extension at 72°C for 10 minutes. The PCR products of CD137L and β‐actin were separated by electrophoresis on a 1% agarose gel containing ethidium bromide (EB) and then photographed under ultraviolet light.

### T cells coculture

To detect CD137L‐induced T cell release of IFN‐γ, CD137L‐expressing 293FT cells and HCC‐827 cells were each cocultured with T cells. First, 1 × 10^5^ transfected (293FT*) or untransfected 293FT cells or HCC‐827 cells and 1 × 10^5^ T cells were separately cultured or cocultured in 96‐well plates in medium alone or media containing anti‐CD3 mAb (1 μg/mL) or anti‐CD137 mAb (10 μg/mL). Control 293FT or HCC‐827 cells were cultured under the same conditions but without adding cDNA. After 48 hours, the supernatant was harvested, and the IFN‐γ concentration was measured. Following the removal of T cells by antihuman CD3 microbeads, the PD‐L1 expression of HCC‐827 cells was analyzed by flow cytometry.

### Detection of supernatant IFN‐γ by flow cytometry

IFN‐γ of the supernatant (15 μL of cell supernatant per sample) was detected using an AimPlex Analyte Kit (AimPlex Biosciences, Inc., Pomona, CA, USA) according to the manufacturer's instructions. The measured IFN‐γ concentrations are presented as the mean ± standard deviation. Statistical analysis was performed using a one‐way analysis of variance (ANOVA) followed by the Dunnett's multiple comparisons test. *P* < 0.05 were considered to indicate a significant difference.

## Results

### PD‐L1 expression by lung cancer cells

**Figure 1:**
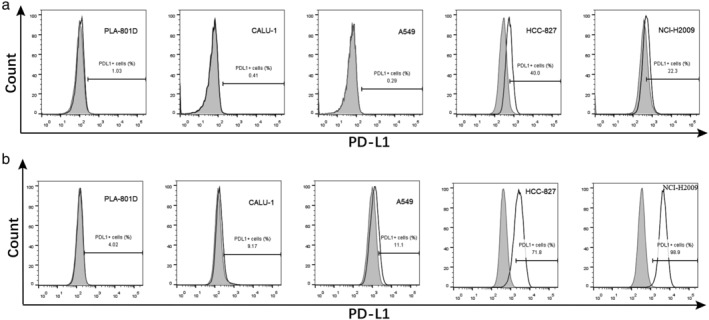
Detected the PD‐L1 expression of human lung cancer cell lines by flow cytometry. (**a**) Of the 13 human lung cancer cell lines, each was stained with PE‐CF594‐labeled mouse antihuman PD‐L1 monoclonal antibody (open histograms) or PE‐CF594‐labeled mouse IgG1, κ isotype control (shaded histograms). (**b**) Each was stained with mouse antihuman PD‐L1 mAb (open histograms) or mouse IgG1, κ isotype control (shaded histograms), followed by staining with a secondary DyLight 488‐conjugated goat antimouse 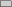 polyclonal Ab.

We first analyzed the PD‐L1 expression in 13 human lung cancer cell lines by flow cytometry. In the present study, we found that all the cell lines expressed PD‐L1 by direct fluorescence staining, including A2 (1.91%), A549 (0.29%), NCI‐H2009 (22.30%), HCC‐827 (40.00%), CALU‐1 (0.41%), NCI‐H2170 (18.1%), NCI‐H1703 (2.15%), PLA‐801D (1.03%), NCI‐H460 (1.20%), NCI‐H661 (1.10%), NCI‐H446 (0.73%), NCI‐H69 (0.90%), NCI‐H209 (3.04%) (Table 1). Compared to fluorescence staining directly, PD‐L1 expression by indirect fluorescence staining was higher, including PLA‐801D (4.02%), A549 (11.1%), CALU‐1 (9.17%), HCC‐827 (71.80%), NCI‐H2009 (98.90%) (Fig 1). Among these, two of five (40%) adenocarcinoma cell lines highly expressed PD‐L1. Additionally, one of two (50%) squamous cell carcinoma cell lines highly expressed PD‐L1, and large cell carcinoma cell lines lowly expressed PD‐L1. Among the three small cell carcinoma cell lines, one had high PD‐L1 expression with a positive rate of 33.3%. The PD‐L1 high expression rate of non‐small cell carcinoma was 40%. Overall, the total PD‐L1 high expression rate of the 13 cell lines was 38.5%. Adenocarcinoma had the highest fluorescence intensity measurements, followed by squamous cell carcinoma, large cell carcinoma, and small cell carcinoma. Thus, the PD‐L1 expression is higher in non‐small cell carcinoma compared with small cell carcinoma.

**Table 1:** The characteristics of the human lung cancer cell lines

Pathological types	Cell lines	PD‐L1 expression before IFN‐γ coculture (%)	PD‐L1 expression after IFN‐γ coculture (%)	After‐before
AC	A2	1.91	51.60	49.69
AC	A549	0.29	44.60	44.31
AC	NCI‐H2009	22.30	77.60	54.70
AC	HCC‐827	40.00	93.00	53.00
AC	CALU‐1	0.41	54.50	54.09
SC	NCI‐H2170	18.10	3.40	−14.70
SC	NCI‐H1703	2.15	31.80	29.65
LCC	PLA‐801D	1.03	34.50	33.47
LCC	NCI‐H661	1.10	24.00	22.90
LCC	NCI‐H460	1.20	55.90	54.70
SCC	NCI‐H209	3.04	11.30	8.26
SCC	NCI‐H69	0.90	7.80	6.90
SCC	NCI‐H446	0.73	43.00	42.27

AC, adenocarcinoma; LCC, large cell carcinoma; SCC, small cell carcinoma; SC, squamous cell carcinoma.

### IFN‐γ is the major cytokine that induces PD‐L1 expression

**Figure 2:**
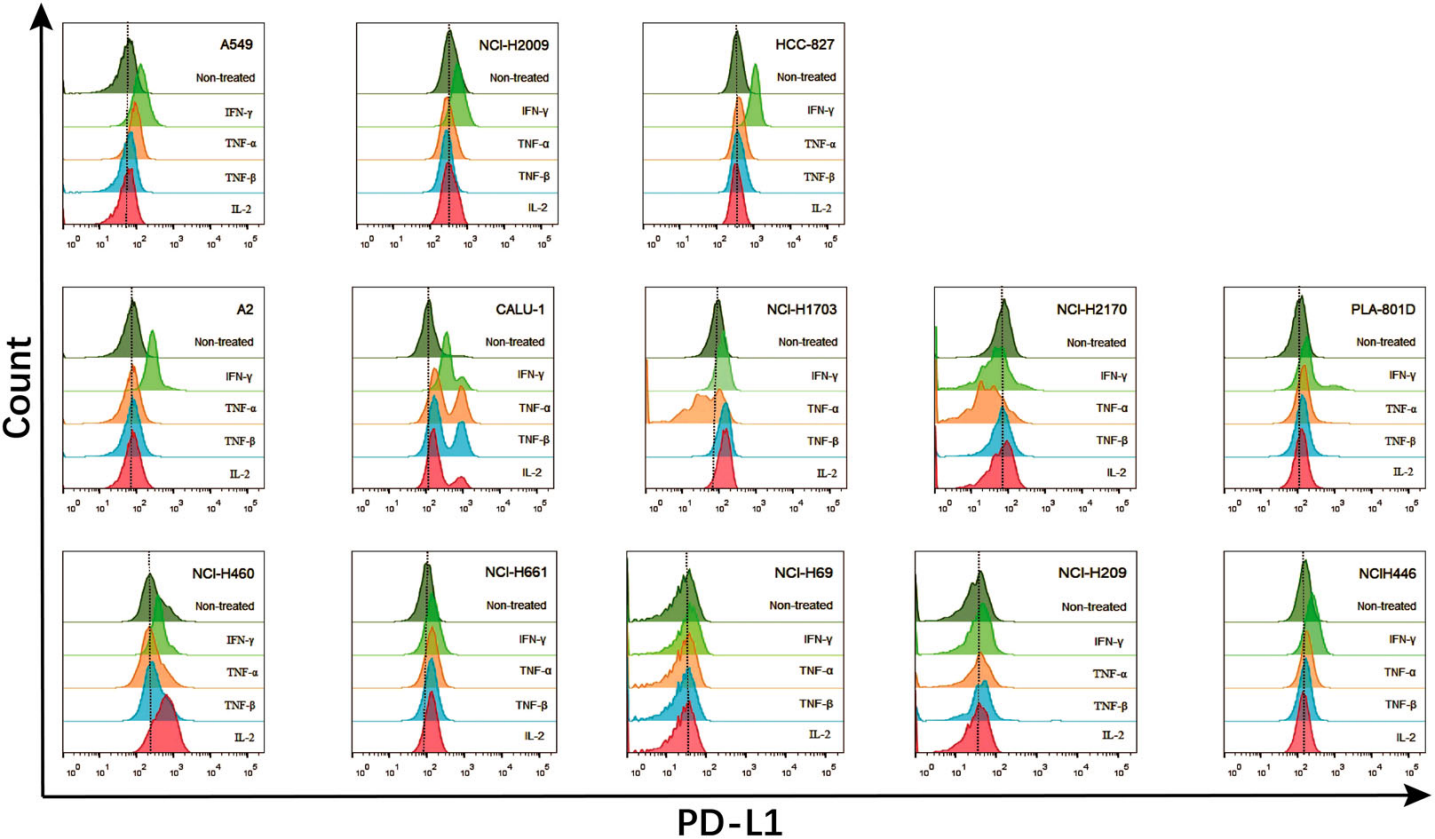
The inducible expression of PD‐L1 in human lung cancer cell lines determined by flow cytometry. Each cell line was cultured in a 24‐well plate. IFN‐γ (100 ng/mL) or TNF‐α (50 ng/mL) or TNF‐β (10 ng/mL) or IL‐2 (100 U/mL) was added per well, respectively and cultured for 72 hours.

IFN‐γ induced the expression of PD‐L1 in almost all tested cell lines, across different pathological types. As shown in Table 1, the highest PD‐L1 levels were induced in five of the adenocarcinoma cell lines, followed by the three large cell cancer cell lines. Among these cell lines, CALU‐1, NCI‐H1703, NCI‐H460, and NCI‐H661 also displayed PD‐L1 expression following stimulation by TNF and IL‐2. Although the other cell lines did not exhibit enhanced PD‐L1 expression following TNF and IL‐2 stimulation, IFN‐γ still induced a relatively high level of PD‐L1 expression in these cells. Our analysis of the PD‐L1 induction by IFN‐γ on cell lines of various pathological types revealed that the effect of this cytokine was the strongest on adenocarcinoma, followed by large cell carcinoma, small cell carcinoma, and squamous cell carcinoma (Fig 2).

### Weak synergistic effect of TNF‐α and IL‐2 with IFN‐γ on inducing PD‐L1 expression

**Figure 3:**
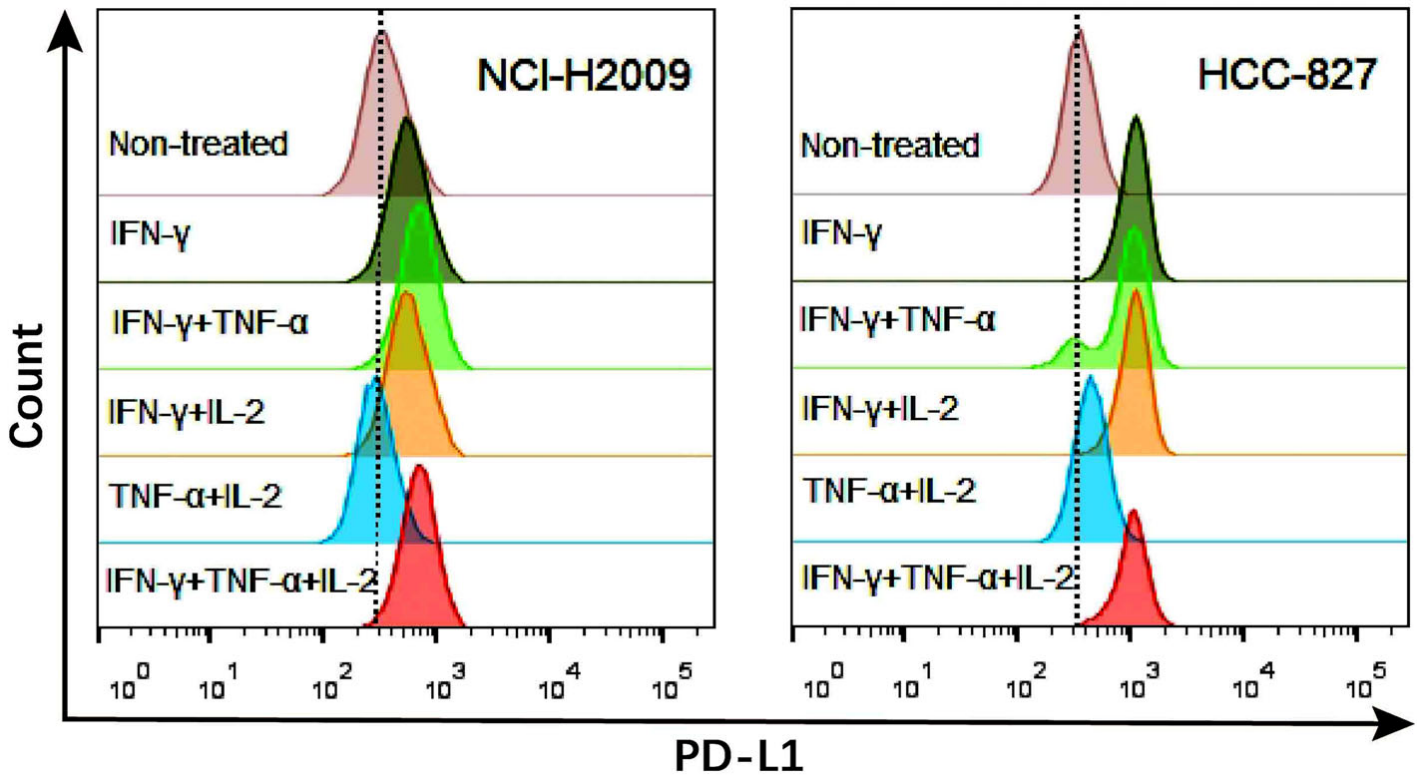
Flow cytometry analysis of TNF‐α, IL‐2 and IFN‐γ synergistically‐induced lung cancer cells to express PD‐L1. NCI‐H2009 and HCC‐827 cells were cultured in 24‐well plates. IFN‐γ (100 ng/mL), TNF‐α (50 ng/mL), IL‐2 (100 U/mL) was added to each well in different combinations, respectively and cultured for 72 hours.

NCI‐H2009 and HCC‐827 cells were used to examine the potential synergistic induction of PD‐L1 by various cytokine combinations. TNF‐α and IL‐2 each had a weak synergistic effect with IFN‐γ on inducing PD‐L1 expression. Notably, the synergistic induction of PD‐L1 by TNF‐α and IL‐2 was significantly weaker than that by IFN‐γ alone (Fig 3).

### Weak CD137L expression by lung cancer cells and IFN‐γ can induce increased expression of CD137L

**Figure 4:**
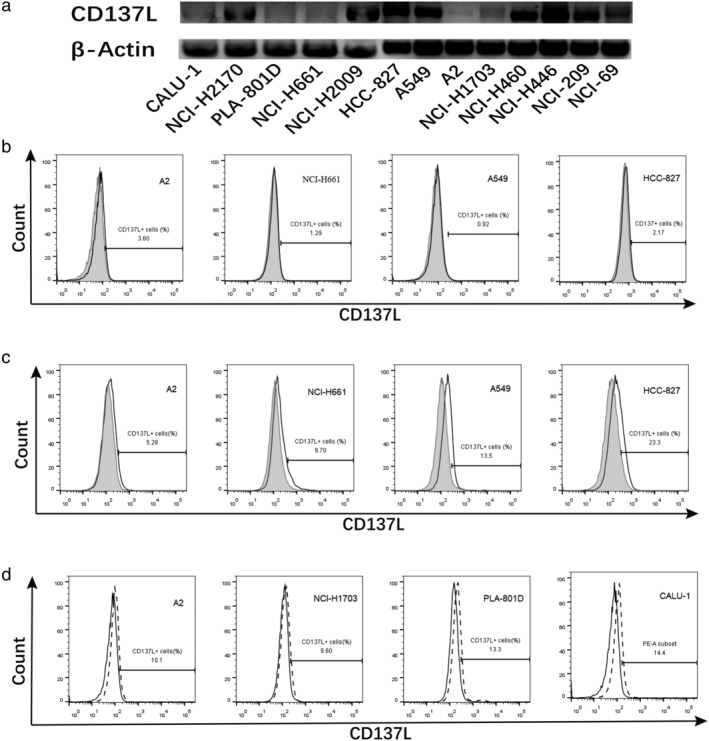
CD137L expression levels were detected by RT‐PCR and flow cytometry with staining directly or indirectly compared to isotype. (**a**) RT‐PCR analysis of lung cancer cell lines CD137L mRNA expression. (**b**) Lung cancer cell lines were each stained with mouse antihuman CD137L mAb (open histograms) or mouse IgG1, κ isotype control (shaded histograms). (**c**) The cells were stained with human CD137 protein (with the Fc region of human IgG) (open histograms) or human B7‐2Ig (shaded histograms), followed by staining with a secondary rabbit antihuman IgG polyclonal Ab. (**d**) The cells were treated with IFN‐γ (dotted line) and control (open histograms) to analyze the CD137L expression by flow cytometry. 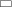 CD137L, 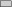 Isotype, 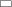 Control and 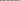 IFN‐γ.

CD137L mRNA levels in cell lines were detected by RT‐PCR. Two adenocarcinoma cell lines (CALU‐1 and A2), one squamous cell carcinoma cell line (NCI‐H1703), and two large cell carcinoma cell lines (NCI‐H661 and PLA‐801D) had no or weak CD137L mRNA expression, whereas CD137L mRNA was highly expressed by the other tested cell lines (Fig 4(a)). A flow cytometry analysis of the lung cancer cells to detect constitutive CD137L protein expression by staining directly revealed that all 13 cell lines weakly expressed this protein (Fig 4(b)). But, CD137L protein expression by staining indirectly was higher (Fig 4(c)). In the five cell lines with weak or no CD137L mRNA expression, the CD137L expression was significantly enhanced in CALU‐1, A2, NCI‐H1703, and PLA‐801D after stimulation with IFN‐γ. Among the other eight cell lines with high CD137L mRNA expression, two cell lines (HCC‐827 and NCI‐H2170) did not exhibit significant changes in the CD137L expression following stimulation with IFN‐γ, whereas the other six cell lines displayed various levels of enhanced CD137L expression (Fig 4(d)).

### CD137L‐expressing lung cancer cells induced T cell secretion of IFN‐γ to promote PD‐L1 expression

**Figure 5:**
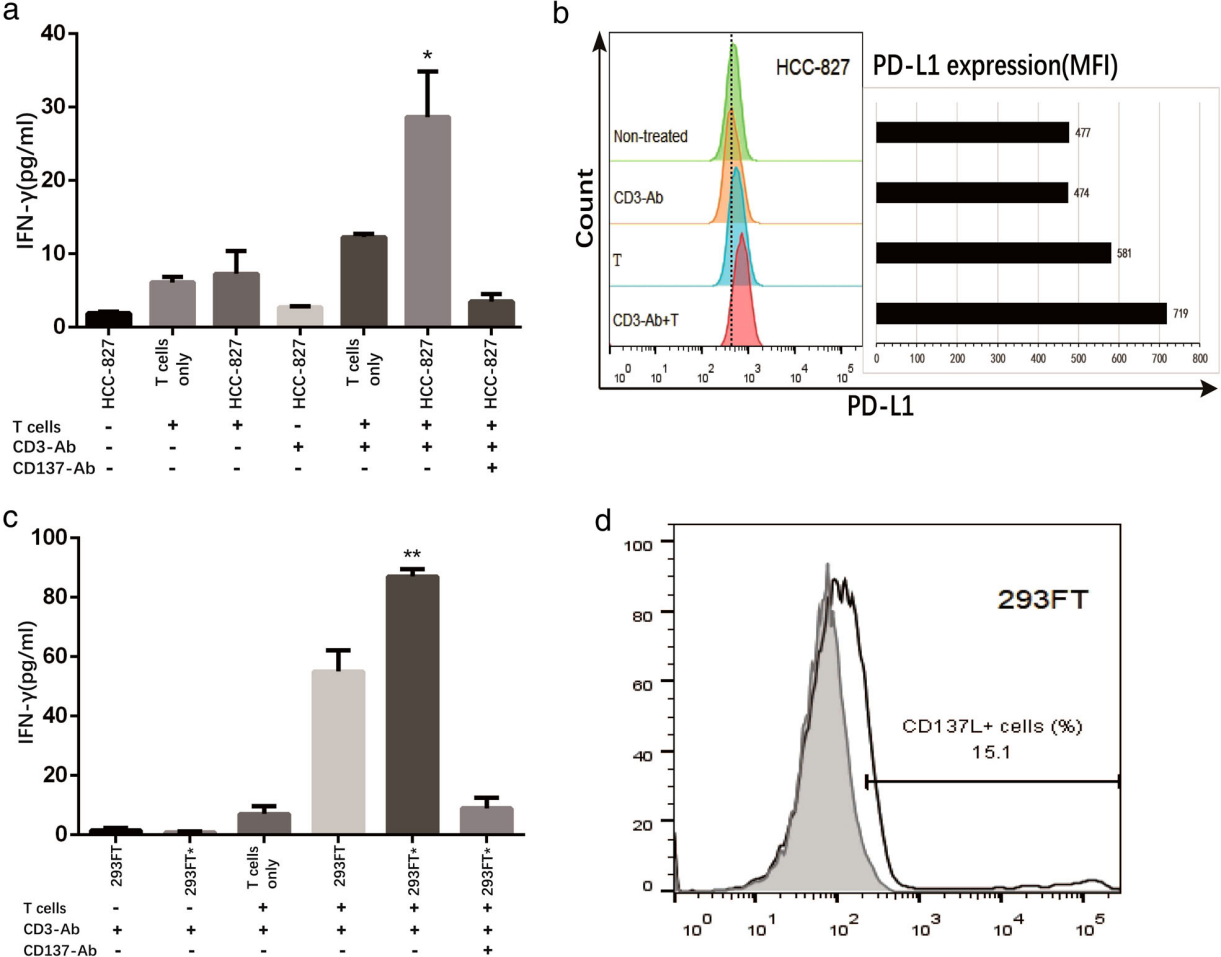
Lung cancer cell lines expressing CD137L induced T cell secretion of IFN‐γ to promote its own PD‐L1 expression. (**a, c**) HCC‐827 or 293FT* (transfected with CD137L plasmid) and T cells were cultured separately or cocultured in 96‐well plates, supplemented with or without anti‐CD3 mAb and anti‐CD137 mAb, and the supernatant was harvested 48 hours later to measure IFN‐γ. (**b**) The PD‐L1 expression of HCC‐827 was determined by flow cytometry after CHCC‐827 cultured alone or cocultured with T cells for 48 hours. (**d**) the 293FT* cells (open histograms) and the control cells nontransfected 293FT (shaded histograms) were detected by flow cytometry. Differences were considered significant at * *P* < 0.05, ** *P* < 0.01.

To evaluate whether CD137L expressed on tumor cells was functionally active, CD137L‐expressing HCC‐827 cells were cocultured with T cells in the presence or absence of soluble antihuman CD3 mAb or antihuman CD137 mAb, and the supernatant IFN‐γ concentration was then measured. Cocultures of HCC‐827 cells with T cells supplemented with anti‐CD3 mAb induced significant increases in the levels of IFN‐γ (28.65 ± 4.41 pg/mL) (*P* < 0.05) compared to absence of anti‐CD3 mAb or HCC‐827. In the presence of anti‐CD137 mAb and anti‐CD3 mAb, T cells cocultured with HCC‐827 cells produced extremely low levels of IFN‐γ (3.52 ± 0.71 pg/mL) (*P* < 0.05) (Fig 5(a)). Flow cytometry analysis of PD‐L1 expression in each group containing HCC‐827 showed that HCC‐827 cells cocultured with T cells and antihuman CD3 mAb had the highest PD‐L1 expression (MFI 719), which was significantly higher than that of containing T cells only group (MFI 581) and containing anti‐CD3 mAb only group (MFI 474) (Fig 5(b)). Interestingly, anti‐CD137 mAb also induced PD‐L1 expression in lung cancer cells and led to a synergistic increase when added with IFN‐γ (data not shown).

To further confirm that the production of IFN‐γ was due to the expression of CD137L by lung cancer cells, transfected 293FT cells (293FT*) were cocultured with T cells. The positive rate of transfection was 15.1% (Fig 5(d)). In the presence of soluble anti‐CD3 mAb, 293FT or 293FT* cells cocultured with T cells produced more IFN‐γ (55.01 ± 5.09 pg/mL and 87.07 ± 1.45 pg/mL, respectively) (*P* < 0.0001) than alone. Furthermore, 293FT* cells produced more IFN‐γ than the 293FT cells (*P* < 0.01). However, in the presence of anti‐CD137 mAb and anti‐CD3 mAb, T cells cocultured with 293FT* cells produced extremely low levels of IFN‐γ (8.95 ± 2.03 pg/mL) (Fig 5(c)).

## Discussion

Many suppressive mechanisms have been recognized, such as the functional impairment or deletion of tumor‐reactive T lymphocytes to suppress T cell function. The recent discovery and definition of the immune escape mechanism in carcinoma development suggested that the mechanism of immune escape leads to local rather than systemic immunosuppression.^10^, ^30^, ^31^ With the progressing study of the PD‐1‐mediated tumor escape mechanism and its successful application in the clinical treatment of tumors, the PD‐1/PD‐L1 pathway has been discovered to be a key adaptive immune escape mechanism of tumors. However, the PD‐L1 expression in lung cancer and its regulation by cytokines in the immunologic tumor microenvironment (TME) is at present not well established. Here, we first examined the PD‐L1 expression status in various established lung cancer cell lines. When the expression of PD‐L1 was detected at the protein level, the total positive rate of PD‐L1 detection was 38.5% across all 13 cell lines. This result demonstrates that the PD‐L1 expression is different among various pathological types. Non‐small cell carcinoma had higher PD‐L1 expression compared with small cell carcinoma, which supports the current indications for anti‐PD‐1/PD‐L1 immunotherapy. Only one out of the three tested small lung cancer cell lines displayed detectable PD‐L1 expression. In the KEYNOTE‐028 study, the objective effective rate of anti‐PD‐1 therapy for small cell lung cancer was 33%,^32^, ^33^ indicating that anti‐PD‐1 immunotherapy may also have value for treating small cell lung cancer in some patients. Our five adenocarcinoma cell lines exhibited the most PD‐L1 expression among the tested cell lines; thus, this pathological type is likely to have the greatest benefit from PD‐L1‐based immunotherapy. Based on our findings regarding the PD‐L1 expression rates and pathological type distribution, the constitutive in vitro expression of PD‐L1 in lung cancer cell lines is basically consistent with the current therapeutic effects of blocking PD‐1/PD‐L1 clinically.

An effective antitumor immune response demands collaboration between CD8^+^ and CD4^+^ T lymphocytes, and CD8^+^ cytotoxic T lymphocytes (CTL) play a central part in antitumor immunity through the production of IFN‐γ, TNF, and granzyme B following ligation of their T cell receptors (TCRs).^34^, ^35^ It has been clearly established that IFN can regulate the PD‐L1 expression in cancer cells and is the mechanism by which carcinoma cells elude being killed by immune cells. In contrast, the other cytokines and molecular pathways that regulate PD‐L1 expression have not been well identified. Here, we further analyzed the potential effects of IFN‐γ, TNF‐α and IL‐2 on PD‐L1 expression. Our results support previous reports that IFN‐γ is the key regulator of PD‐L1 expression. Among of all tested cell lines, the five adenocarcinoma cell lines showed the most obvious induction of PD‐L1 expression. Not surprisingly, the intensity of PD‐L1 expression varied among the tested lung cancer types. The IFN‐γ induction efficiency was the strongest in adenocarcinoma. Studies have reported that PD‐L1 expression in adenocarcinoma was higher compared with squamous cell carcinoma in tissue.^12^, ^36^, ^37^, ^38^, ^39^, ^40^ Thus, this study further confirmed that IFN‐γ is one of the most important cytokines for PD‐L1 expression in lung cancer.

An IFN‐induced upregulation of PD‐L1 expression in carcinoma cell lines was first reported by Dong *et al*.^41^ and tumor cell‐associated PD‐L1 expression was later found to increase the apoptosis of antigen‐specific T lymphocytes in vitro. This mechanism represents a localized immune deficiency that allows tumors to escape, known as “adaptive immune tolerance”.^39^, ^40^ Both TNF‐α and IL‐2 are critical components of an inflammatory TME, which may contribute to regulating PD‐L1 expression. It was previously reported that TNF‐α and IFN‐γ synergistically induced PD‐L1 expression in hepatocellular carcinoma cells.^42^ However, in our study on 13 lung cancer cell lines, these two cytokines in isolation have limited regulatory effects on PD‐L1 expression. The cells treated with TNF or IL‐2 had only a minor synergistic effect on the IFN‐γ‐induced expression of PD‐L1. Our results indicate that IFN‐γ is the core cytokine that regulates PD‐L1 expression in the lung carcinoma microenvironment.

Anti‐CD137 mAb stimulates CD8^+^ T cells to produce IFN‐γ.^43^ CD137 signaling can enhance T cell function and is also dependent on other TNF‐receptor associated factors (TRAFs) that associate TNFRs with NF‐κB and on stress kinase signaling pathways.^34^, ^35^ In vivo, there were also reports showing a strong IFN‐γ production when CD137 signaling was stimulated by using a CD137 agonist.^44^, ^45^ CD137L provides a CD28‐independent signal resulting in cell division, and delivered systemically increases CD8^+^ T cell expansion in comparison to CD4^+^ T cell expansion.^46^, ^47^ IFN‐γ is mainly derived from CD8^+^ T cells and is the key effective factor of active CD8^+^ T cells. The constitutive expression of functional CD137L has been reported on various carcinoma cells, along with the suggestion that CD137L expressed by carcinoma cells may significantly affect the outcome of T cell‐tumor cell interactions.^26^ In the present work, we found that except for two large cell lung cancer cell lines that did not express or only weakly expressed CD137L, most lung cancer cell lines tested in our study highly expressed CD137L at the mRNA level, which is in accordance with a previous report.^26^ However, when the CD137L expression in lung carcinoma cells was analyzed by flow cytometry, all 13 lung cancer cell lines showed weak expression at the protein level. Interestingly, we found that IFN‐γ could also upregulate CD137L expression in lung carcinoma cell lines across various pathological types, with a particularly strong effect in the adenocarcinoma lines. The 293FT cells transfected with CD137L clearly showed costimulating effects on T lymphocytes. A cytokine analysis of the activated T lymphocytes revealed that CD137L costimulation markedly enhanced the production of IFN‐γ by T lymphocytes. The HCC‐827, in which the CD137L expression is low at the protein level, had an improved PD‐L1 expression following coculture with T cells, most likely mediated by IFN‐γ. Additionally, the CD137L expression on HCC‐827 cells significantly induced IFN‐γ production because this could be completely inhibited by treatment with anti‐CD137 mAb. It has been suggested that CD137 is sensitive to stimulation by CD137L,^48^, ^49^ so even these low expression levels might be sufficient to trigger CD137 signaling upon interaction with CD137L. More interestingly, we found that anti‐CD137 mAb can also induce the expression of PD‐L1 in lung cancer cells and synergize with IFN‐γ. The relevant mechanism needs further study.

Based on our findings, we propose that CD137L could upregulate PD‐L1 expression on lung cancer via a feedback loop involving IFN‐γ production by T cells. This regulatory loop probably exists in the TME. The finding that CD137L expressed by tumor cells promoted T cells to secrete IFN‐γ, which subsequently enhanced the PDL1 and CD137L expression in lung carcinoma cells simultaneous. CD137L, as a positive feedback of costimulatory molecule, not only positively regulates the immune function of T cells, but also initiates the negative immune regulation mechanism. This may represent a potential mechanism of tumor escape from cytotoxic T lymphocytes. From this point of view, our results may support the combined therapeutic value of anti‐CD137 mAb and anti‐PD‐1/PD‐L1 mAb, which, in addition to being assessed in several preclinical studies, has been investigated in clinical trials.^20^

In conclusion, we found that CD137L mRNA was widely expressed in lung cancer cell lines whereas levels of protein expression were generally low. However, the low level of CD137L protein was still enough to induce T cells to produce IFN‐γ that subsequently increased PD‐L1 expression. The CD137L‐induced negative immune regulation may represent a mechanism of immune escape.

## Disclosure

No authors report any conflict of interest.

## References

[1] Zou W , Wolchok JD , Chen L . PD‐L1 (B7‐H1) and PD‐1 pathway blockade for cancer therapy: Mechanisms, response biomarkers, and combinations. Sci Transl Med 2016; 8 (328): 328rv4. 10.1126/scitranslmed.aad7118 PMC4859220 26936508

[2] Sharma P , Allison JP . The future of immune checkpoint therapy. Science 2015; 348 (6230): 56–61. 25838373 10.1126/science.aaa8172

[3] Topalian SLDCG , Pardoll DM . Immune checkpoint blockade: A common denominator approach to cancer therapy. Cancer Cell 2015; 27: 450–61. 25858804 10.1016/j.ccell.2015.03.001 PMC4400238

[4] DM P . The blockade of immune checkpoints in cancer immunotherapy. Nat Rev Cancer 2012; 12: 252–64. 22437870 10.1038/nrc3239 PMC4856023

[5] Iwai Y , Hamanishi J , Chamoto K , Honjo T . Cancer immunotherapies targeting the PD‐1 signaling pathway. J Biomed Sci 2017; 24 (1): 26. 28376884 10.1186/s12929-017-0329-9 PMC5381059

[6] Sznol M , Chen L . Antagonist antibodies to PD‐1 and B7‐H1 (PD‐L1) in the treatment of advanced human cancer. Clin Cancer Res 2013; 19 (5): 1021–34. 23460533 10.1158/1078-0432.CCR-12-2063 PMC3702373

[7] Shimoji M , Shimizu S , Sato K *et al* Clinical and pathologic features of lung cancer expressing programmed cell death ligand 1 (PD‐L1). Lung Cancer 2016; 98: 69–75. 27393509 10.1016/j.lungcan.2016.04.021

[8] Iwai Y , Ishida M , Tanaka Y , Okazaki T , Honjo T , Minato N . Involvement of PD‐L1 on tumor cells in the escape from host immune system and tumor immunotherapy by PD‐L1 blockade. Proc Natl Acad Sci U S A 2002; 99 (19): 12293–7. 12218188 10.1073/pnas.192461099 PMC129438

[9] Hirano F , Kaneko K , Tamura H *et al* Blockade of B7‐H1 and PD‐1 by monoclonal antibodies potentiates cancer therapeutic immunity. Cancer Res 2005; 65 (3): 1089–96. 15705911

[10] Taube JM , Anders RA , Young GD *et al* Colocalization of inflammatory response with B7‐h1 expression in human melanocytic lesions supports an adaptive resistance mechanism of immune escape. Sci Transl Med 2012; 4 (127): 127ra37. 10.1126/scitranslmed.3003689 PMC3568523 22461641

[11] Tumeh PC , Harview CL , Yearley JH *et al* PD‐1 blockade induces responses by inhibiting adaptive immune resistance. Nature 2014; 515 (7528): 568–71. 25428505 10.1038/nature13954 PMC4246418

[12] Azuma K , Ota K , Kawahara A *et al* Association of PD‐L1 overexpression with activating EGFR mutations in surgically resected nonsmall‐cell lung cancer. Ann Oncol 2014; 25 (10): 1935–40. 25009014 10.1093/annonc/mdu242

[13] Freeman GJ , Long AJ , Iwai Y *et al* Engagement of the PD‐1 immunoinhibitory receptor by a novel B7 family member leads to negative regulation of lymphocyte activation. J Exp Med 2000; 192 (7): 1027–34. 11015443 10.1084/jem.192.7.1027 PMC2193311

[14] Li N , Wang J , Zhang N *et al* Cross‐talk between TNF‐alpha and IFN‐gamma signaling in induction of B7‐H1 expression in hepatocellular carcinoma cells. Cancer Immunol Immunother 2018; 67 (2): 271–83. 29090321 10.1007/s00262-017-2086-8 PMC11028210

[15] Zhao Q , Xiao X , Wu Y *et al* Interleukin‐17‐educated monocytes suppress cytotoxic T‐cell function through B7‐H1 in hepatocellular carcinoma patients. Eur J Immunol 2011; 41 (8): 2314–22. 21674477 10.1002/eji.201041282

[16] Curiel TJ , Wei S , Dong H *et al* Blockade of B7‐H1 improves myeloid dendritic cell‐mediated antitumor immunity. Nat Med 2003; 9 (5): 562–7. 12704383 10.1038/nm863

[17] Karakhanova S , Meisel S , Ring S , Mahnke K , Enk AH . ERK/p38 MAP‐kinases and PI3K are involved in the differential regulation of B7‐H1 expression in DC subsets. Eur J Immunol 2010; 40 (1): 254–66. 19830728 10.1002/eji.200939289

[18] Kil SH , Estephan R , Sanchez J *et al* PD‐L1 is regulated by interferon gamma and interleukin 6 through STAT1 and STAT3 signaling in cutaneous T‐cell lymphoma. Blood 2017; 130: 1458.

[19] Matta BM , Raimondi G , Rosborough BR , Sumpter TL , Thomson AW . IL‐27 production and STAT3‐dependent upregulation of B7‐H1 mediate immune regulatory functions of liver plasmacytoid dendritic cells. J Immunol 2012; 188 (11): 5227–37. 22508931 10.4049/jimmunol.1103382 PMC3564546

[20] Chester C , Sanmamed MF , Wang J , Melero I . Immunotherapy targeting 4‐1BB: Mechanistic rationale, clinical results, and future strategies. Blood 2018; 131 (1): 49–57. 29118009 10.1182/blood-2017-06-741041

[21] Kwon BS , Weissman SM . cDNA sequences of two inducible T‐cell genes. Proc Natl Acad Sci U S A 1989; 86 (6): 1963–7. 2784565 10.1073/pnas.86.6.1963 PMC286825

[22] Niu L , Strahotin S , Hewes B *et al* Cytokine‐mediated disruption of lymphocyte trafficking, hemopoiesis, and induction of lymphopenia, anemia, and thrombocytopenia in anti‐CD137‐treated mice. J Immunol 2007; 178 (7): 4194–213. 17371976 10.4049/jimmunol.178.7.4194 PMC2770095

[23] Dubrot J , Milheiro F , Alfaro C *et al* Treatment with anti‐CD137 mAbs causes intense accumulations of liver T cells without selective antitumor immunotherapeutic effects in this organ. Cancer Immunol Immunother 2010; 59 (8): 1223–33. 20336294 10.1007/s00262-010-0846-9 PMC11030554

[24] Sica G , Chen LP . Modulation of the immune response through 4‐1BB. Adv Exp Med Biol 2000; 465: 355–62. 10810639 10.1007/0-306-46817-4_30

[25] Vinay DS , Kwon BS . Role of 4‐1BB in immune responses. Semin Immunol 1998; 10 (6): 481–9. 9826581 10.1006/smim.1998.0157

[26] Salih HR , Kosowski SG , Haluska VF *et al* Constitutive expression of functional 4‐1BB (CD137) ligand on carcinoma cells. J Immunol 2000; 165 (5): 2903–10. 10946324 10.4049/jimmunol.165.5.2903

[27] Chen S , Lee L , Fisher TS *et al* Combination of 4‐1BB agonist and PD‐1 antagonist promotes antitumor effector/memory CD8 T cells in a poorly immunogenic tumor model. Cancer Immunol Res 2015; 3 (2): 149–60. 25387892 10.1158/2326-6066.CIR-14-0118

[28] Wei H , Zhao L , Hellstrom I , Hellstrom KE , Guo Y . Dual targeting of CD137 co‐stimulatory and PD‐1 co‐inhibitory molecules for ovarian cancer immunotherapy. Oncoimmunology 2014; 3 (4): e28248. 10.4161/onci.28248 PMC4063147 25050196

[29] Dai M , Yip Y , Hellstrom I , Hellstrom KE . Curing mice with large tumors by locally delivering combinations of immunomodulatory antibodies. Clin Cancer Res 2015; 21 (5): 1127–38. 25142145 10.1158/1078-0432.CCR-14-1339 PMC4336234

[30] Sanmamed MF , Chen L . A paradigm shift in cancer immunotherapy: From enhancement to normalization. Cell 2018; 175 (2): 313–26. 30290139 10.1016/j.cell.2018.09.035 PMC6538253

[31] Chen L , Han X . Anti‐PD‐1/PD‐L1 therapy of human cancer: Past, present, and future. J Clin Invest 2015; 125: 3384–91. 26325035 10.1172/JCI80011 PMC4588282

[32] Carbone DP , Reck M , Paz‐Ares L *et al* First‐line nivolumab in stage IV or recurrent non‐small‐cell lung cancer. N Engl J Med 2017; 376 (25): 2415–26. 28636851 10.1056/NEJMoa1613493 PMC6487310

[33] Ott PA , Bang YJ , Piha‐Paul SA *et al* T‐cell–inflamed gene‐expression profile, programmed death ligand 1 expression, and tumor mutational burden predict efficacy in patients treated with Pembrolizumab across 20 cancers: KEYNOTE‐028. J Clin Oncol 2019; 37 (4): 318–27. 30557521 10.1200/JCO.2018.78.2276

[34] Li G , JC B , Kotani H *et al* 4‐1BB enhancement of CART function requires NF‐kappaB and TRAFs. JCI Insight 2018; 3: 121322. 30232281 10.1172/jci.insight.121322 PMC6237232

[35] Jang IK , Lee ZH , Kim YJ , Kim SH , Kwon BS . Human 4‐1BB (CD137) signals are mediated by TRAF2 and activate nuclear factor‐kappa B. Biochem Biophys Res Commun 1998; 242 (3): 613–20. 9464265 10.1006/bbrc.1997.8016

[36] Mu CY , Huang JA , Chen Y , Chen C , Zhang XG . High expression of PD‐L1 in lung cancer may contribute to poor prognosis and tumor cells immune escape through suppressing tumor infiltrating dendritic cells maturation. Med Oncol 2011; 28 (3): 682–8. 20373055 10.1007/s12032-010-9515-2

[37] Konishi J , Yamazaki K , Azuma M , Kinoshita I , Dosaka‐Akita H , Nishimura M . B7‐h1 expression on non‐small cell lung cancer cells and its relationship with tumor‐infiltrating lymphocytes and their PD‐1 expression. Clin Cancer Res 2004; 10 (15): 5094–100. 15297412 10.1158/1078-0432.CCR-04-0428

[38] Kitazono S , Fujiwara Y , Tsuta K *et al* Reliability of small biopsy samples compared with resected specimens for the determination of programmed death‐ligand 1 expression in non–small‐cell lung cancer. Clin Lung Cancer 2015; 16 (5): 385–90. 25937270 10.1016/j.cllc.2015.03.008

[39] Ota K , Azuma K , Kawahara A *et al* Induction of PD‐L1 expression by the EML4‐ALK oncoprotein and downstream signaling pathways in non‐small cell lung cancer. Clin Cancer Res 2015; 21 (17): 4014–21. 26019170 10.1158/1078-0432.CCR-15-0016

[40] D'Incecco A , Andreozzi M , Ludovini V *et al* PD‐1 and PD‐L1 expression in molecularly selected non‐small‐cell lung cancer patients. Br J Cancer 2015; 112 (1): 95–102. 25349974 10.1038/bjc.2014.555 PMC4453606

[41] Dong H , Strome SE , Salomao DR *et al* Tumor‐associated B7‐H1 promotes T‐cell apoptosis: A potential mechanism of immune evasion. Nat Med 2002; 8 (8): 793–800. 12091876 10.1038/nm730

[42] Löffek S . Transforming of the tumor microenvironment: Implications for TGF‐β inhibition in the context of immune‐checkpoint therapy. J Oncol 2018; 2018: 1–9. 10.1155/2018/9732939 PMC6304495 30631358

[43] Shuford WW , Klussman K , Tritchler DD *et al* 4‐1BB costimulatory signals preferentially induce CD8+ T cell proliferation and lead to the amplification in vivo of cytotoxic T cell responses. J Exp Med 1997; 186: 47–55. 9206996 10.1084/jem.186.1.47 PMC2198949

[44] Lee SW , Salek‐Ardakarni S , Mittler RS , Croft M . Hypercostimulation through 4‐1BB distorts homeostasis of immune cells. J Immunol 2009; 182 (11): 6753–62. 19454670 10.4049/jimmunol.0803241 PMC2713069

[45] Wang J , Zhao W , Cheng L *et al* CD137‐mediated pathogenesis from chronic hepatitis to hepatocellular carcinoma in hepatitis B virus‐transgenic mice. J Immunol 2010; 185 (12): 7654–62. 21059892 10.4049/jimmunol.1000927 PMC3601909

[46] DeBenedette MA , Shahinian A , Mak TW , Watts TH . Costimulation of CD28‐ T lymphocytes by 4‐1BB ligand. J Immunol 1997; 158 (2): 551–9. 8992967

[47] Wang C , Lin GHY , McPherson AJ , Watts TH . Immune regulation by 4‐1BB and 4‐1BBL: Complexities and challenges. Immunol Rev 2009; 229: 192–215. 19426223 10.1111/j.1600-065X.2009.00765.x

[48] Vanamee ES , Faustman DL . Structural principles of tumor necrosis factor superfamily signaling. Sci Signal 2018; 11 (511): eaao4910. 10.1126/scisignal.aao4910 29295955

[49] Zapata JM , Perez‐Chacon G , Carr‐Baena P *et al* CD137 (4‐1BB) signalosome: Complexity is a matter of TRAFs. Front Immunol 2018; 9: 2618. 30524423 10.3389/fimmu.2018.02618 PMC6262405

